# Endothelial biomarkers in patients with familial Mediterranean fever associated vascular disease and vasculopathy

**DOI:** 10.1186/1546-0096-13-S1-O16

**Published:** 2015-09-28

**Authors:** Y Sargsyan, A Sargsyan

**Affiliations:** 1 Yerevan State Medical University, Yerevan, Armenia; 2 Yerevan State Medical University, Internal Medicine, Yerevan, Armenia

## Objectives

FMF is an autosomal recessive disease, caused by mutations in the MEFV gene, encoding the pyrin protein associated with the interleukin-1β related inflammation cascade. In FMF overproduction of IL-1β and uncontrolled TNF-α release has been documented. These cytokines mediate activation of endothelial cells causing sustained inflammatory response and endothelial cell dysfunction[^1^]. Vascular injuries are frequently seen in FMF patients although the exact mechanisms are not well understood. We design this study to determine the role of endothelial biomarkers in pathogenesis of vasculopathy in FMF.

## Methods

The study cohort included 50 FMF patients with vascular involvement (VI, mean age 39.7±12, male/female 23/27) - group 1, and 52 FMF patients without VI (31.6±11, 32/20) - group 2. A total of 50 healthy subjects between 16-67 years of age were enrolled as controls. All patients diagnosed in accordance with Tel-Hashomer criteria. VI was determined by physical examination, lab tests and instrumental modalities (ECG, Doppler echocardiography, CT-scan and MRI). The patients group with VI included 30 FMF patients with coronary heart disease (2 of them with myocardial infarction), 14 with pulmonary hypertension, 2 with stroke, 1 with poliarteritis nodosa, 1 with Henoch-Schonlein purpura, 1 with livedoid vasculopathy and 1 with Raynaud's phenomenon. Laboratory tests, including leucocyte count, erythrocyte sedimentation rate, fibrinogen, C-reactive protein, serum amyloid-A, nitric oxide and endothelin-1 were carried out on all patients in attack free period. CRP was determined by immunoturbidimetric method and SAA by ELISA. Serum NO and plasma ET-1 levels were measured by Griess reaction and enzyme immunoassay, respectively. Normal values of NO and ET-1 in our laboratory were <20μmol/L and 7.2±4pg/ml, respectively.

## Results

Fibrinogen and leucocyte count were normal in patient groups (mean±SD, group 1 vs group 2) 4.46±1.1g/L vs 3.57±1 and 8.5±2.4 (x10^9^)g/L vs 7.4±1.6, whereas ESR was increased 30.3±18.7 mm/h vs 19±12.6. Mean CRP and SAA were significantly higher in group 1 than in group 2 (21.06±16.8 mg/L vs 12,5±12.1, t=2.94, p<0.01; 30.56±63.3 mg/L vs 10.47±32,79, t=2,0013, P<0,05, respectively). The mean level of NO was lower in group 2 than in controls (3.12±0.75μmol/l vs 8.79±2.72 μmol/l, t=14.2, p<0.0001) and in group 1 than in group 2 (2.79±0.7 μmol/l vs 3.12±0.75 μmol/l, t=2.3, p<0.05), though it was within normal ranges. Although there were no correlations between NO, fibrinogen, leucocytes, ESR and SAA in both patients group, there were negative correlations between NO and CRP (r=-0.338, p=0,016, group 1; r=-0,398, p=0,003, group 2). There was a significant difference in ET-1 levels between the controls and the patient group 2 (7.8.±1.99 pg/ml vs 16.2±7.32 pg/ml, t=7.97, p<0.0001) and between the patient group 2 and 1 (16.2±7.32 pg/ml vs 23.13±14.8 pg/ml, t=2.98, p<0.05). Also ET-1 levels showed positive correlations with CRP in patient groups (r=0,585, p=0,000, group 1 and r=0,285, p=0,041, group 2), and with SAA in group 1 (r=0,459, p=0,001). No correlations were found between ET-1, fibrinogen, leucocytes and ESR.

## Conclusions

Endothelial biomarkers were found abnormal in patients with FMF. We have shown that levels of NO in FMF patients were normal, but lower compared with healthy controls. Expression of NO is induced drastically by multiple stimuli- interferon-α, tumor necrosis factor-α and IL-1β that induce iNOS (inducible nitric oxide synthase) expression in macrophages, leucocytes and vascular endothelial cells [1]. NO inhibits the biochemical effect of ET-1, however, ET-1 induces iNOS expression in endothelial cells. Impaired NO production may contribute to the pathogenesis of vascular injury in FMF.

We have revealed an increased ET-1 level in the patients with FMF associated vascular disease. We have also demonstrated that there were significant correlations between ET-1 levels and inflammatory markers, such as CRP and SAA, in the patients with FMF associated vascular disease. We conclude that continuous low-grade inflammation in FMF may cause vascular injury and endothelial dysfunction that may lead to vasoconstriction and ischemic complications in FMF patients with the development of cardiovascular, pulmonary, neurological and other vascular events. Last but not least, endothelin-1 may have a role in the pathogenesis of FMF associated vascular disease.

## Acknowledgements

We thank chief of the laboratory Anahit Davtyan for collaboration.
